# Double rarity: malignant masquerade biliary stricture in a situs inversus totalis patient

**DOI:** 10.1186/s12893-021-01155-w

**Published:** 2021-03-21

**Authors:** K. Eitler, Z. Mathe, V. Papp, A. Zalatnai, A. Bibok, P. A. Deak, L. Kobori, G. Telkes

**Affiliations:** 1 Department of Transplantation and Surgery, Semmelweis University, VIII. Baross u.23, Budapest, 1082 Hungary; 2 1st. Department of Surgery and Interventional Gastroenterology, Semmelweis University, Budapest, Hungary; 3 1st. Department of Pathology and Experimental Cancer Research, Semmelweis University, Budapest, Hungary

**Keywords:** Situs inversus, Common bile duct, Obstructive jaundice, Perihilar cholangiocarcinoma, Klatskin tumor

## Abstract

**Background:**

Situs inversus totalis is a rare anatomical variation of both the thoracic and the abdominal organs. Common bile duct strictures can be caused by malignant and benign diseases as well. 7–18% of the latter ones are 'malignant masquerade’ cases, as pre-operative differentiation is difficult.

**Case presentation:**

We present the case of a 68y male patient with known situs inversus totalis and a recent onset of obstructive jaundice caused by a malignant behaving common bile duct stricture. Technically difficult endoscopic retrograde cholangiopancreatography, brush cytology, magnetic resonance cholangiopancreatography, endoscopic ultrasound, and percutaneous transhepatic drainage with stent implantation were performed for proper diagnosis. Cholecystectomy, common bile duct resection with hilar lymphadenectomy, and hepatico-jejunostomy have been performed following multidisciplinary consultation. The final histology report did not confirm any clear malignancy, the patient is doing well.

**Conclusion:**

In situs inversus patients, both diagnostic and therapeutic procedures can lead to various difficulties. Benign biliary strictures are frequently misdiagnosed preoperatively as cholangiocellular carcinoma. Surgery is usually unavoidable, involving a significant risk of complications. The co-existence of these two difficult diagnostic and therapeutic features made our case challenging.

**Supplementary Information:**

The online version contains supplementary material available at 10.1186/s12893-021-01155-w.

## Background

Situs inversus totalis (SIT) is a rare congenital abnormality characterized by a mirror-image transposition of both the abdominal and the thoracic organs. Its incidence accounts for 1/8000 to 1/25,000 of the normal population [1]. This condition might cause difficulties during diagnostic and also therapeutic procedures [2]. Because of its rarity, surgeons usually do not have much surgical experience with these patients. Stricture or stenosis of the extrahepatic biliary structures can be caused by malignant and benign diseases as well. Pre-operative differentiation of the benign strictures and the malignant tumours of the biliary structures is difficult because of the lack of non-invasive diagnostic procedures, and also benign lesions can be clinically present as malignant [3]. Still, about 50% of patients with suspected perihilar cholangiocarcinoma are subjected to resection nowadays without a histologically proven diagnosis [4].

## Case presentation

**Fig. 1:**
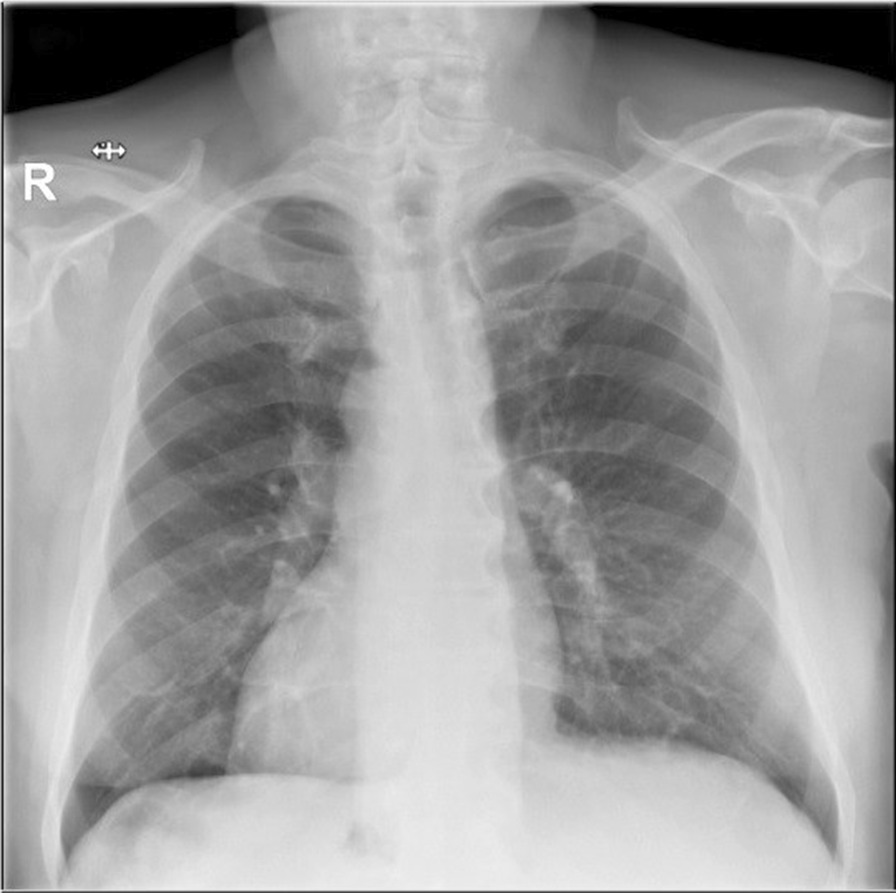
Chest X-ray of the patient with dextrocardia

A 68-year-old male patient was referred to our tertiary surgical institute having obstructive jaundice, pruritus and subcutaneous haematomas for two months. At the age of 10, chest X-ray revealed the situs inversus totalis (Fig. 1) for the first time. He was on warfarin due to chronic atrial fibrillation.

**Fig. 2:**
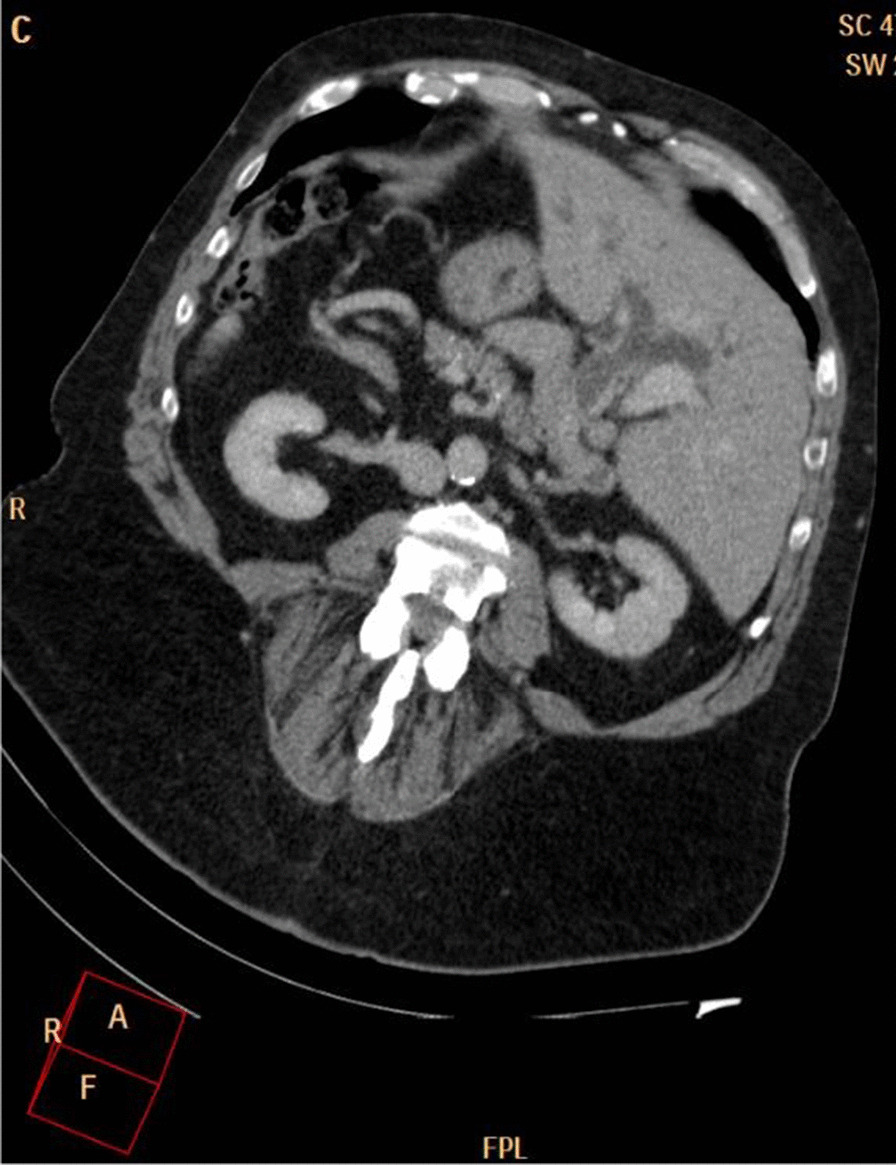
Preoperative CT scan: the liver is located on the left side, the intra- and extrahepatic bile ducts are dilated

**Fig. 3:**
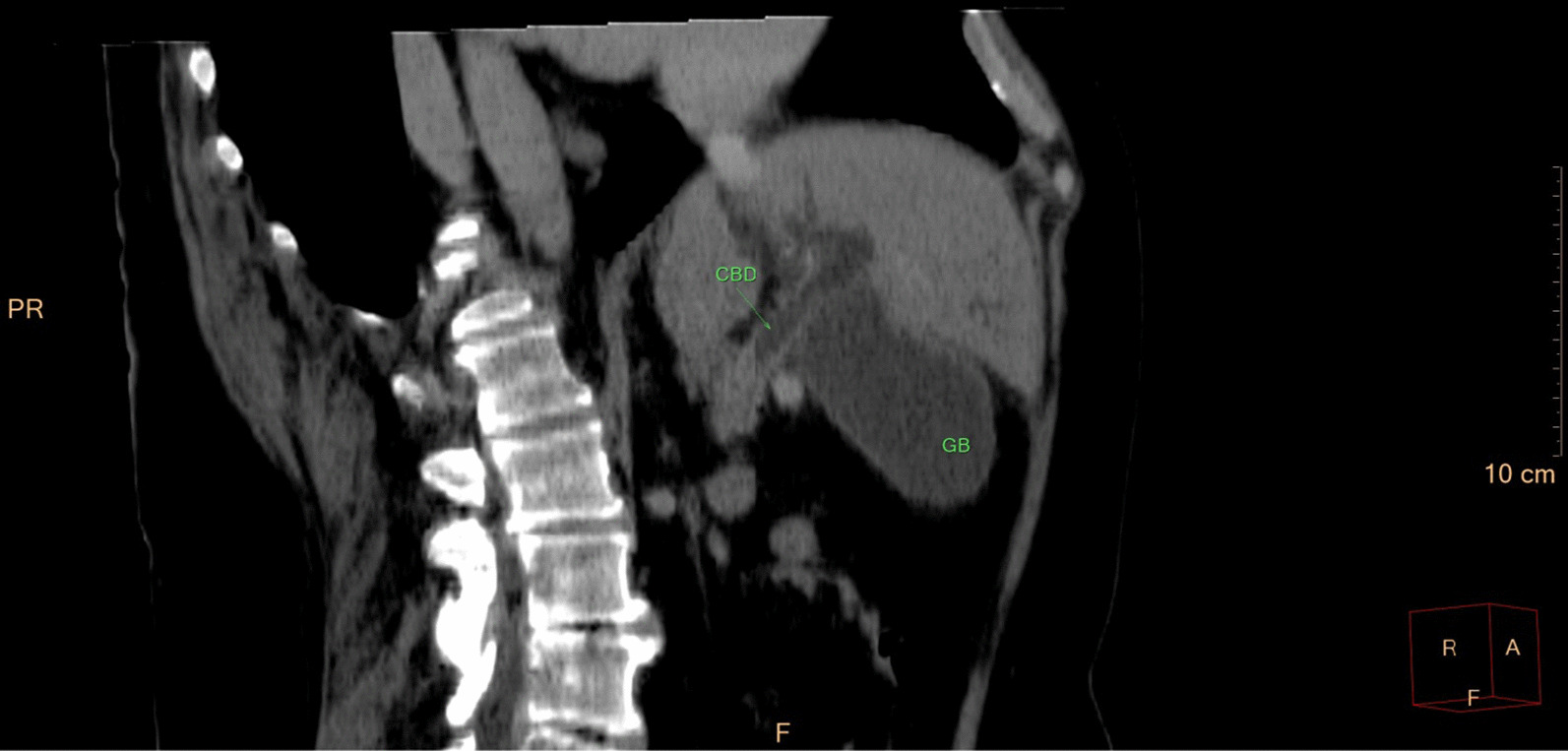
CT – pre-stenotic common bile duct dilatation

**Fig. 4:**
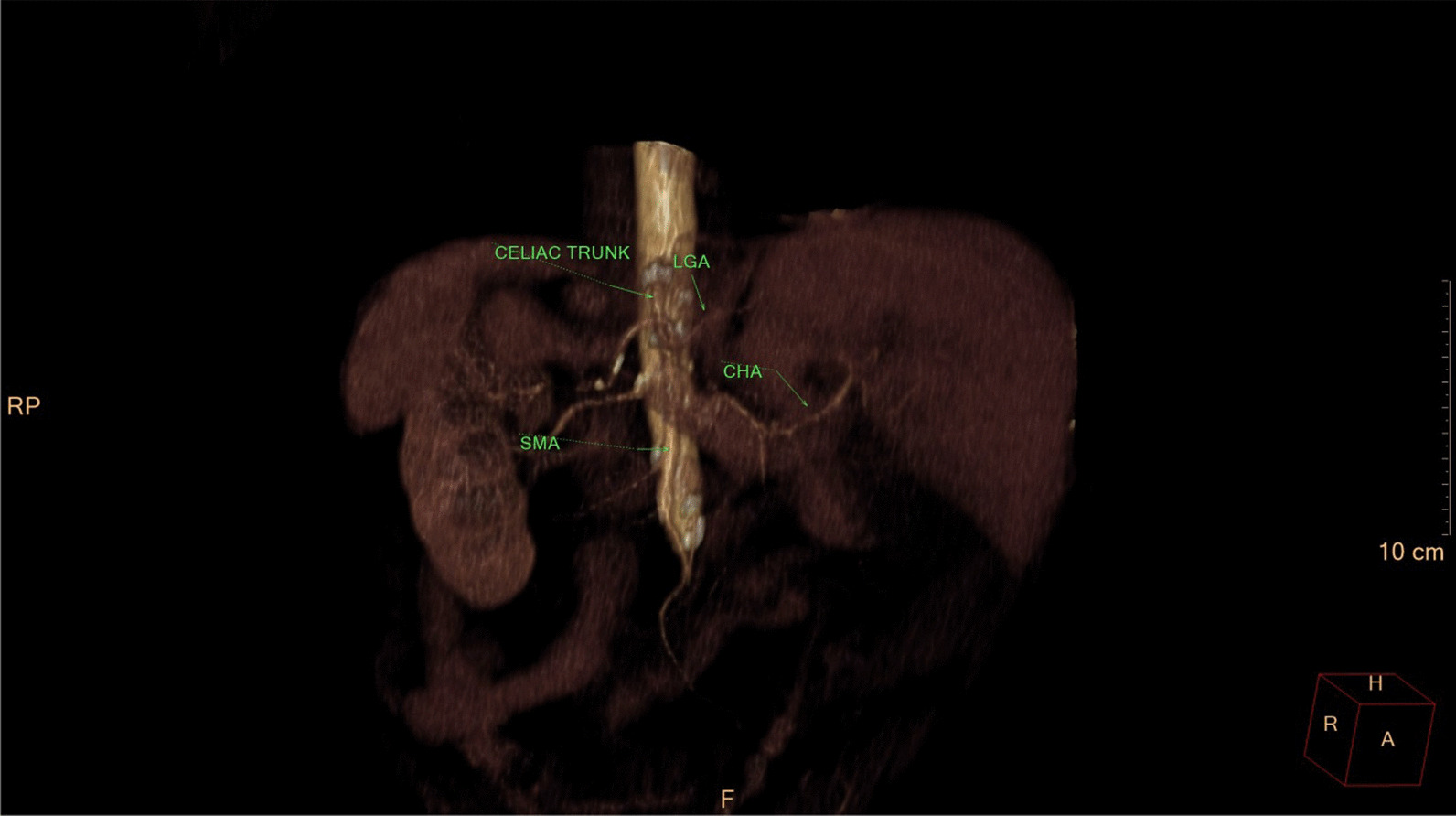
CT volume rendered 3D reconstruction. Variant anatomy of hepatic artery: replaced CHA, originating from the SMA and there is an accessory LHA from LGA

**Fig. 5:**
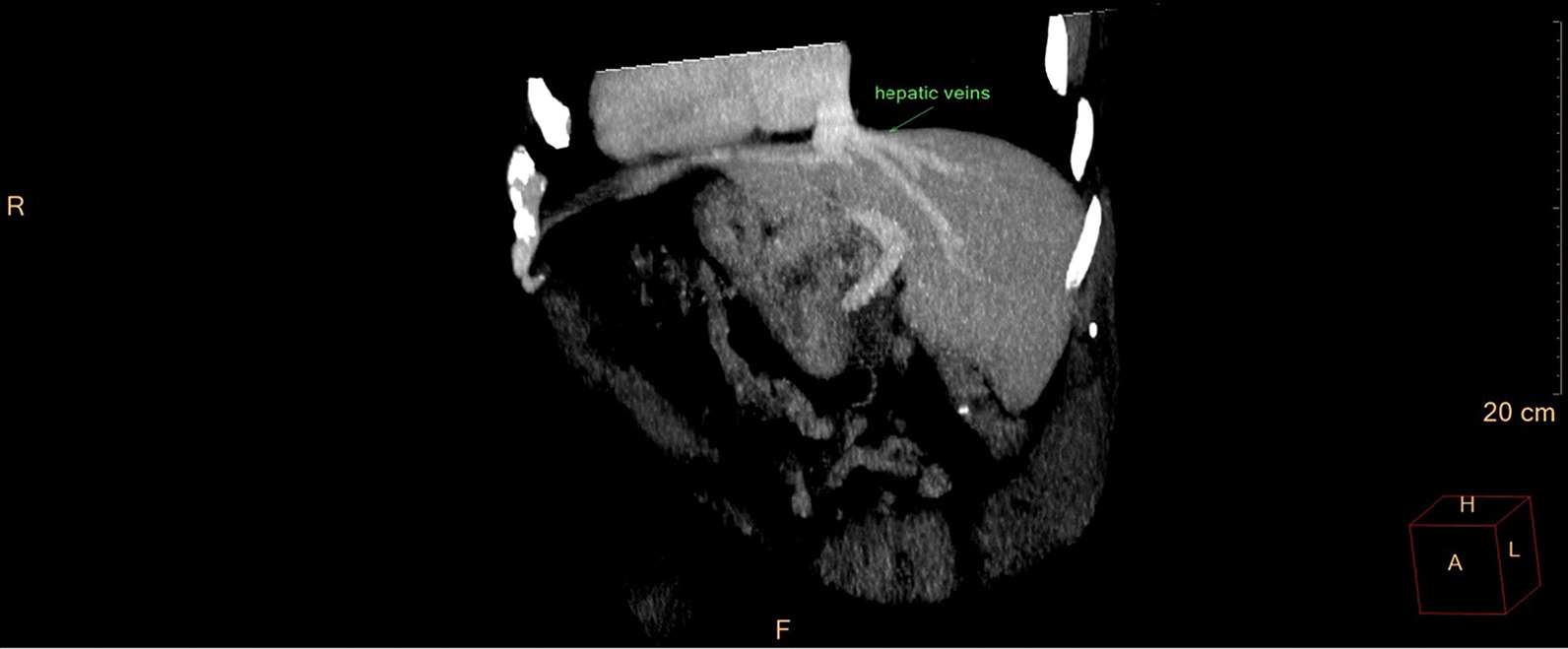
CT—maximum intensity projection—all hepatic veins drain directly into the atrium

**Fig. 6:**
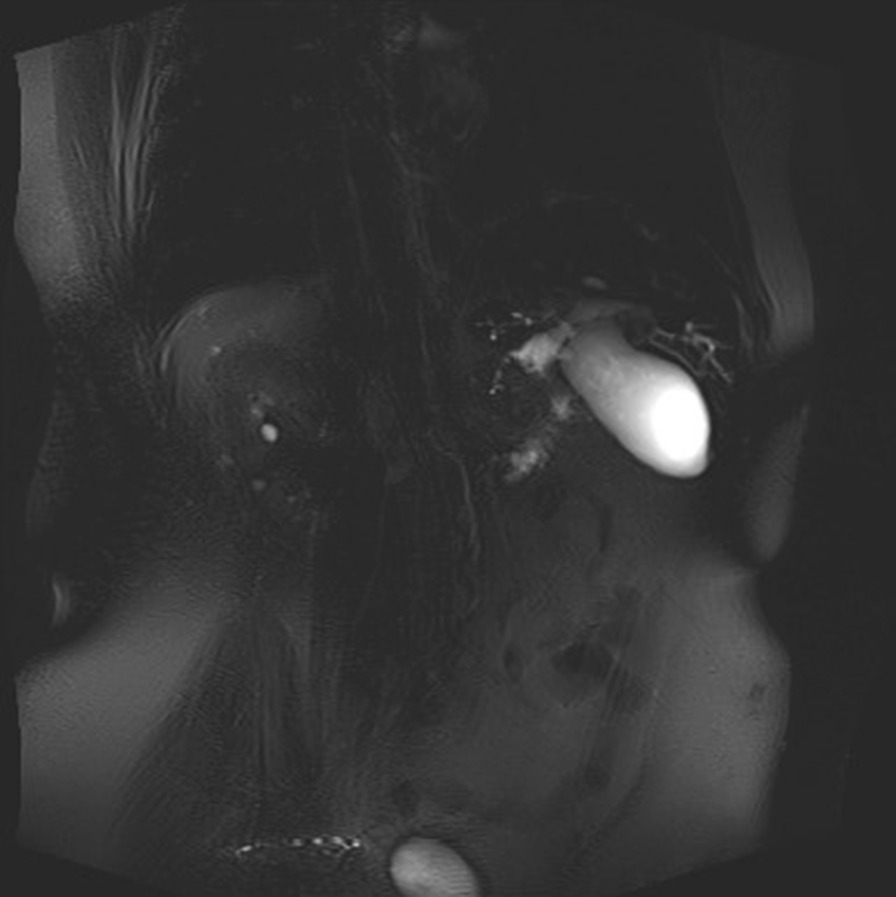
MRI- pre-stenotic common bile duct dilatation

**Fig. 7:**
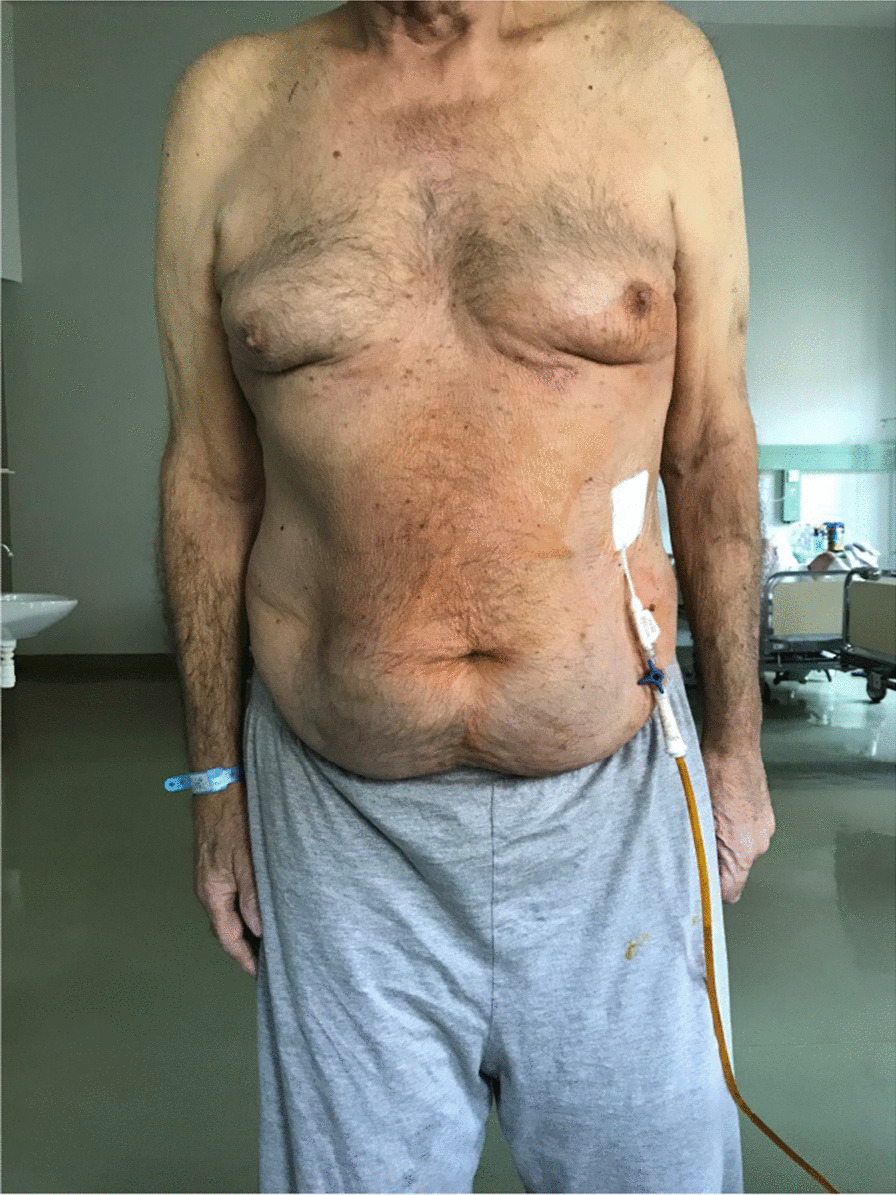
PTD to the anatomically right side of the liver, in this case it was located on the left side of the patient

**Fig. 8:**
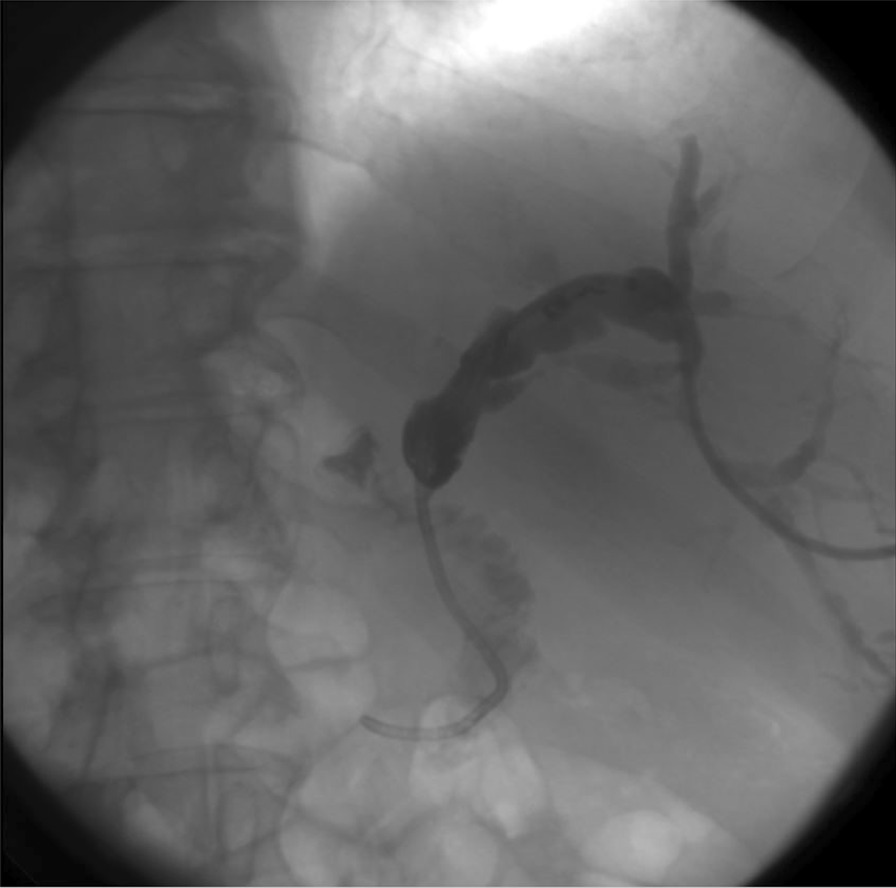
PTD-cholangiogram: The suspicion of Bismuth-Corlette stage II Klatskin tumour arose because a subocclusive stenosis of the extrahepatic bile duct was proven

**Fig. 9:**
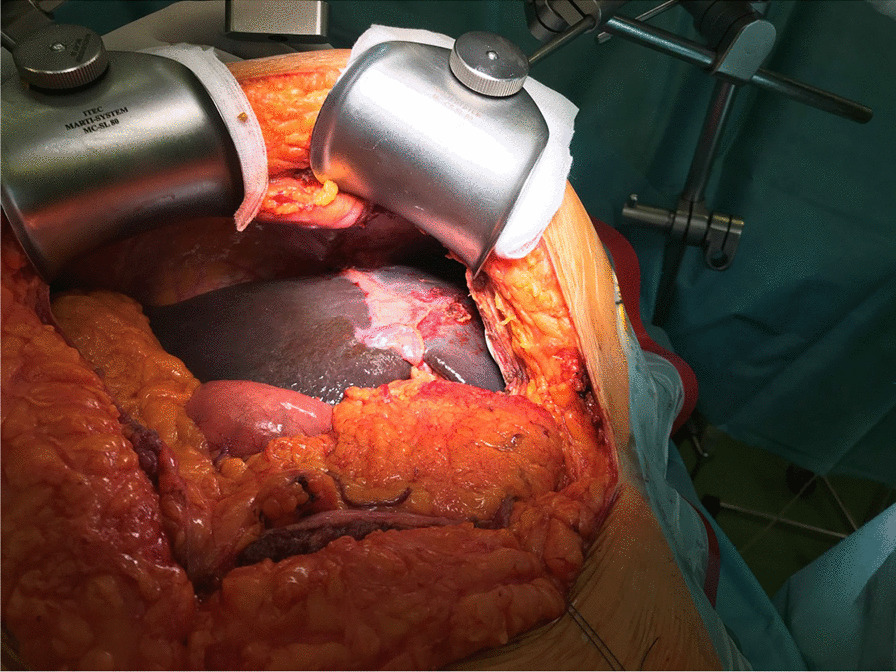
Intraabdominal situation. The liver is situated on the left, the stomach is on the right side

**Fig. 10:**
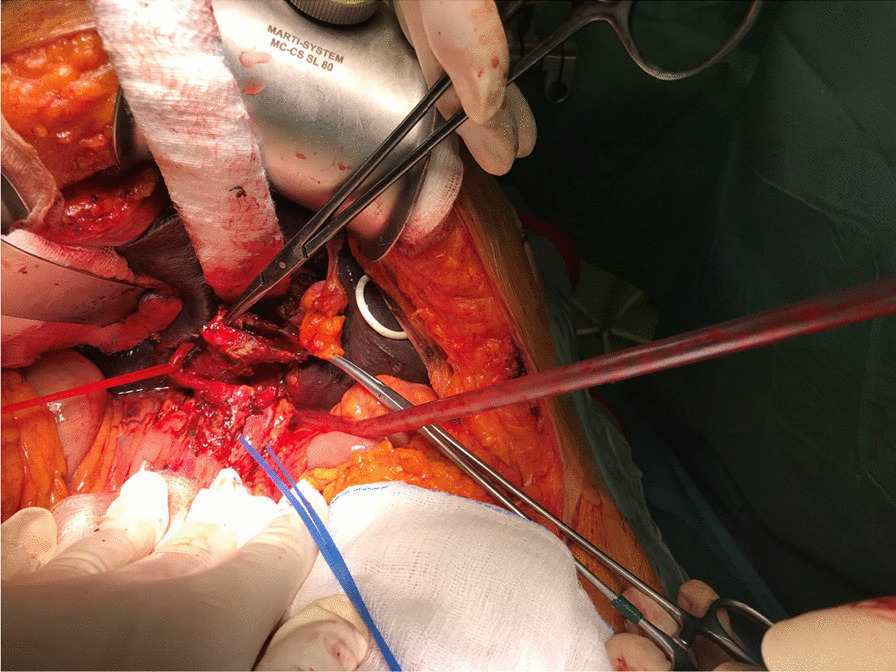
Intraoperative situation. Hepatic artery (red) and portal vein (blue)

**Fig. 11:**
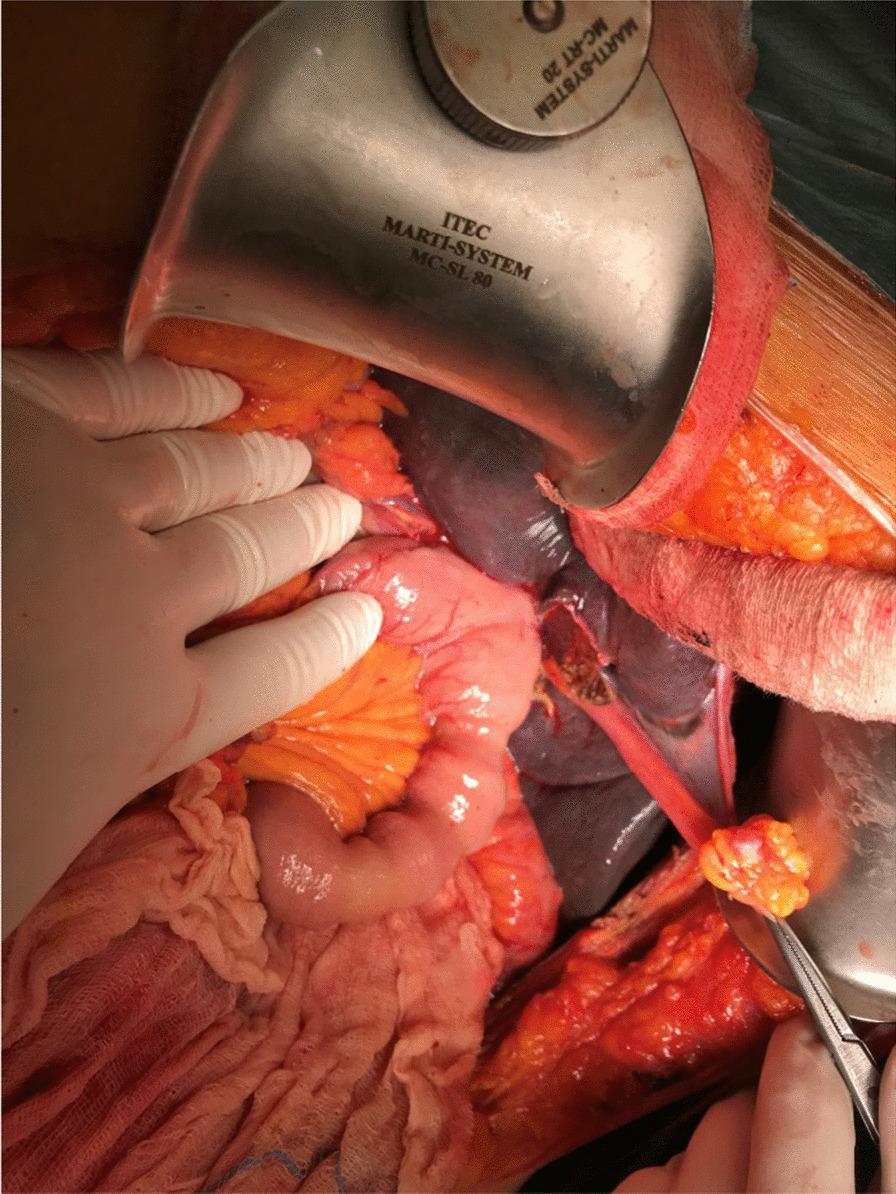
After reconstruction: mirrored hepaticojejunostomy

**Fig. 12:**
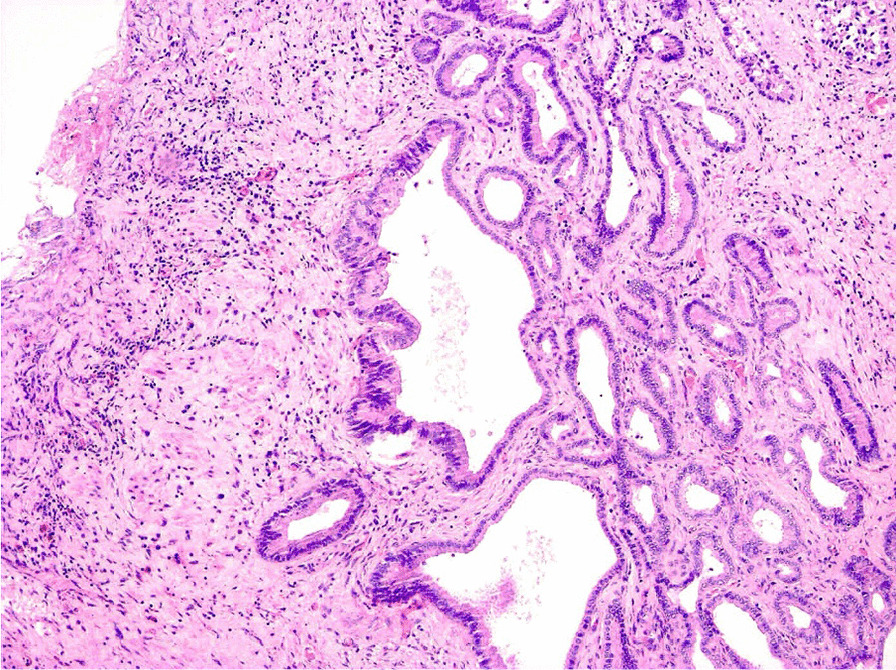
Histology. Haematoxylin–eosin stain (×100). Proliferating glands of the bile ducts are shown. Clear malignancy is not appearing in this sample, some dysplasia can be seen on the left side and the stromal tissue is strongly fibrotic

**Fig. 13:**
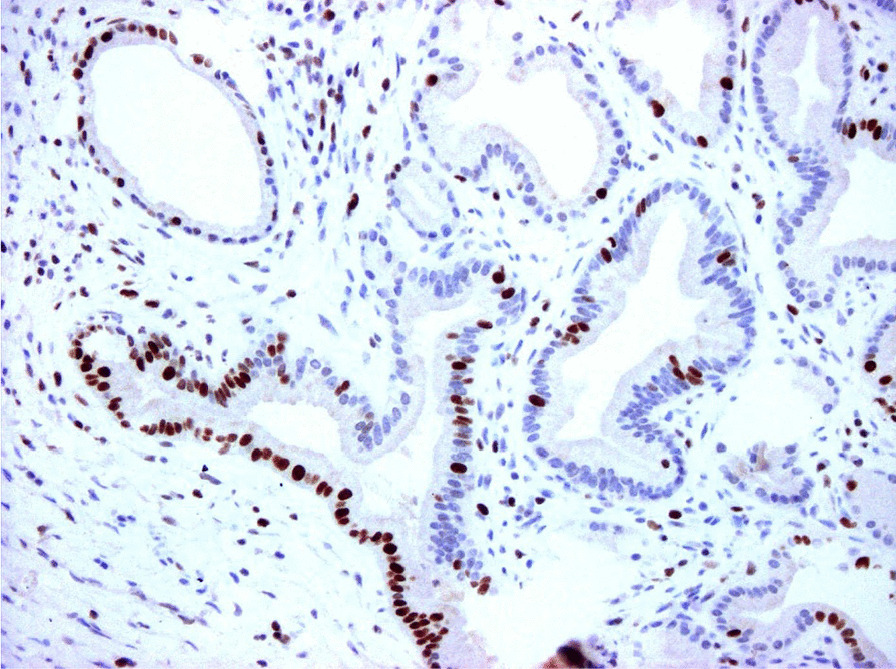
Histology with EZH-2 immunohistochemistry (×200). Most of the proliferating bile ducts are EZH-2-negative, mild positivity is shown at some places (dysplastic cells)

Eventually, liver enzymes, bilirubin and alkaline phosphatase levels were elevated. Current computer tomography (CT) confirmed a normal size liver located on the left side; the intra- and extrahepatic bile ducts and the common bile duct (CBD) were dilated (Fig. 2, 3). Vascular abnormalities were also detected: the “replaced” common hepatic artery (CHA) originates from the superior mesenteric artery (SMA, Michel’s classification type 9), (Fig. 4) venous drainage from the liver was ensured by an independent vein, connecting straight to the right atrium of the heart (Fig. 5). Venous drainage from the lower part of the body and abdominal organs was ensured by the inferior caval vein located on the right side of the aorta. The first attempt of endoscopic retrograde cholangiopancreatography (ERCP) was not successful due to the different anatomical situation. Magnetic resonance cholangiopancreatography (MRCP) confirmed the dilatation of the extra- and intrahepatic bile ducts (Fig. 6). Endoscopic ultrasound (EUS) did not find any clear sign of malignancy neither in the biliary tree nor in the pancreas. The second attempt at ERCP verified a duplex stenosis of the CBD affecting both its intrapancreatic and hilar section. Biliary stent placement was unsuccessful. Brush cytology sampling was performed: from the intrapancreatic section of the CBD, malignancy could not be proved, and the sample from the hilar part was uninterpretable. Percutaneous transhepatic drainage (PTD) was introduced to one of dilated bile ducts of the’right’ lobe on the left side of the liver, and biliary stent placement was successful this time: the bilirubin level of the patient started to decrease (Fig. 7, 8). Surgical consultation and a multidisciplinary oncology team assumed a Bismuth-Corlette stage II Klatskin tumour (cholangiocarcinoma) and suggested surgery. Distal resection of the common bile duct, cholecystectomy and hilar lymphadenectomy have been performed (Figs. 9, 10) at our institution. We performed hepatico-jejunostomy to restore the biliary tract (Fig. 11). The intraoperative frozen section initially showed tumour cells from the distal part of the CBD. Then, at the second attempt, after re-resecting 2 more millimeters from the CBD, it came back tumour-free. The resection was R0—no cancer cells were seen microscopically. The final histological analysis of the specimen did not confirm our diagnosis: it did not prove clear malignancy, but some epithelial dysplasia was discoverable with haematoxylin and eosin (H&E) staining (Fig. 12). Some intensive positivity was seen in the biliary structures (Fig. 13) by enhancer of zeste homologue 2 (EZH2) immunohistochemical demonstration.

After the surgery, the patient had a minor wound infection treated by bedside wound opening (Clavien-Dindo classification stage I); he did not have any other surgical complications, and was discharged after 21 days. A multidisciplinary oncology team discussed the case again after the procedure, and no oncological treatment was considered necessary. After 3 months of the surgery, the control CT scan was completely tumour-free (Additional file 1).

## Discussion

**Fig. 14:**
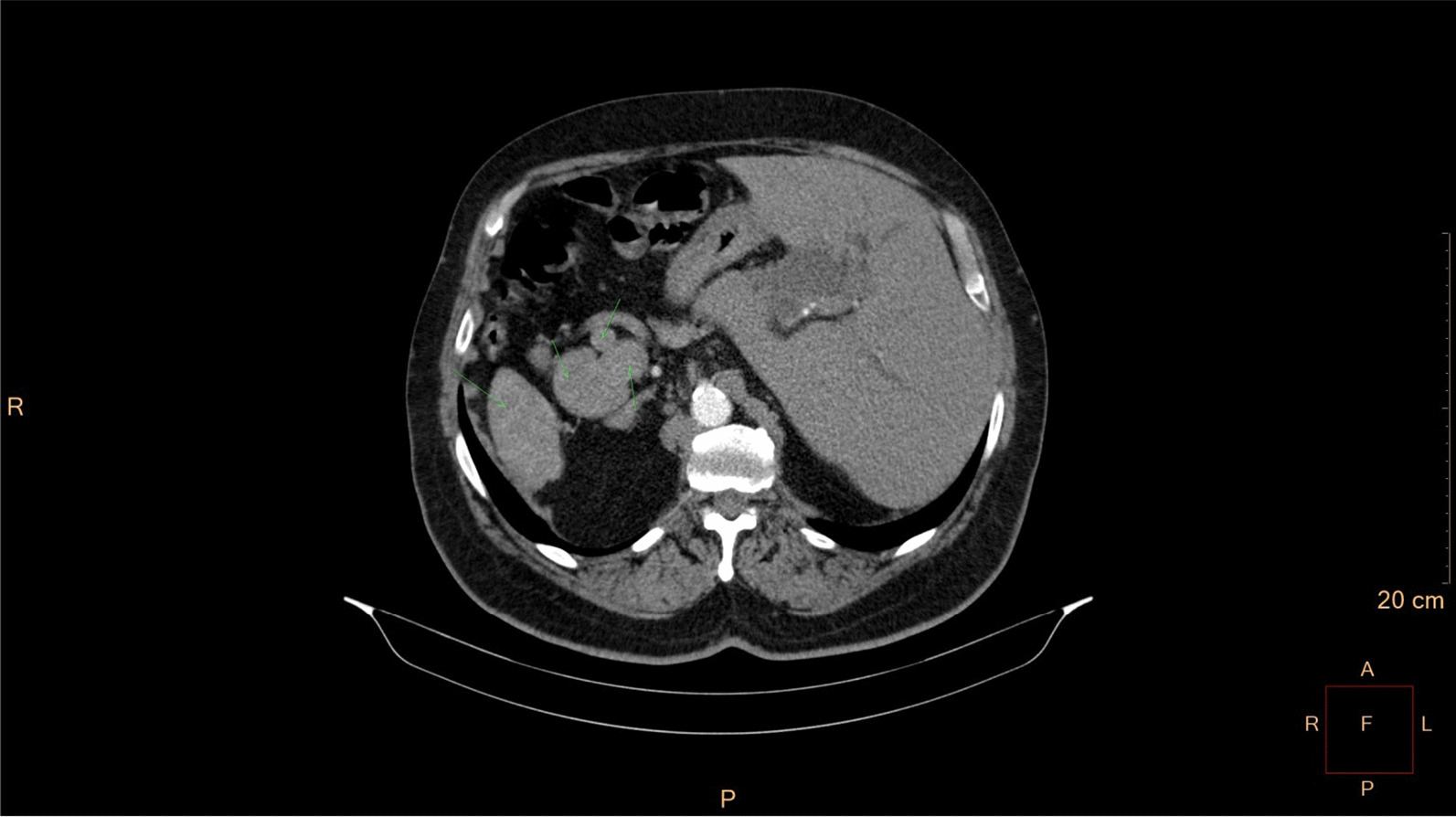
Multiple splenules—multiple spleens often related to SIT

Situs inversus totalis is a rare asymptomatic congenital anomaly. During embryonic development, the embryonic midgut normally rotates counter-clockwise at 270°, but in case of SIT, it performs this rotation clockwise. Polysplenia is frequent in SIT patients as it was observed in our case as well (Fig. 14). Most of the time, situs inversus itself is asymptomatic: it is revealed by chance. In situs inversus patients, both diagnostic and therapeutic procedures can cause various difficulties. The inverse location of the organs might lead to misdiagnosis as a result of the false projection of the pain to the opposite side of the body, and sometimes it requires technical changes in diagnostic procedures [5]. In endoscopic and minimally invasive surgical procedures, a’mirror image technique ‘ can be used; the patient and the equipment are placed on the opposite side and the procedure itself is performed inversely, with the operating surgeon standing on the left side of the patient [6]. In laparoscopic procedures, careful consideration of trocar positioning is necessary [7]. However, for right-handed surgeons, this can cause some difficulties: it can be uncomfortable to handle the instruments with their left hand or the pedals with their left foot [8]. Therefore, the most important factor is the careful exploration of anatomical variations—especially the vascular anomalies that often occur—by using preoperative imaging techniques.

In the condition of situs inversus, surgery can be challenging as a result of the different anatomical situation. Although situs inversus totalis itself is not a serious hazard to normal health and long-term survival, the early diagnosis of this condition is crucial, especially when the patient’s condition requires surgical intervention [9].

In our case, the right-handed operating surgeon was standing in his standard position: on the right side of the patient. Because of its rarity and special nature, surgical patients with situs inversus may require more flexibility and creativity from the surgical team; however, many cases that have been reported in the literature—including our case as well- could have been treated more successfully [5].

Patients with situs inversus may have a higher risk for cancer. While SIT itself is not a premalignant condition, numerous types of malignancies have been reported in the literature, e.g.: colorectal carcinoma, renal cell carcinoma, gastric cancer, lung cancer, gallbladder cancer, and ovarian embryonal cancer. The incidence of any type of malignancies is sporadic in SIT, the association between the two conditions could be a rare coincidence [10].

In the pathogenesis of SIT, intracellular motorproteins such as KIF3 play important roles. In case of non-functional KIF3, the cell-adhesion factors N-cadherin and β-catenin are not transported to the cell surface. These proteins are involved in the development and progression of cancer [11, 12].

Perihilar cholangiocarcinoma (also known as Klatskin’s tumour) is a rare gastrointestinal malignancy (representing 3% of all types of gastrointestinal tumours), originating from the intra- or extrahepatic bile duct epithelium [13]. Still, it is the most frequent cause of common bile duct stricture. It is usually classified according to the ductal involvement of the tumour. This classification was described by Bismuth et al. in 1988 [14], and it is the standard for decision making on resecability. There are very few documented cases of cholangiocellular carcinoma presented in situs inversus patients in the literature; only 6 cases have been reported [15].

The rate of benign strictures misdiagnosed preoperatively as cholangiocellular carcinoma is high, varying data can be found in the literature: about 7–18%, even up to 50% [4, 16, 17]. The term’malignant masquerade ‘ was first used in 1985 by Hadjis et al.: they described the malignant-behaving, but histologically proven benign bile duct strictures by this expression, the lesions of which cannot be distinguished by clinical or imaging features [18]. Although major hepatobiliary resections have become safer in the past decades, and these patients are now referred to tertiary referral centers, morbidity (30–50%) and mortality (5%) rates of such of operations are still high [19]. Non-malignant causes of biliary obstructions are mostly due to inflammation, further defined as fibroinflammatory processes [20]. Lymphoplasmacytic sclerosing pancreatitis and cholangitis frequently involves the head of the pancreas and the distal common bile duct, and can form masses that can resemble malignancies. Diffuse fibroinflammatory lymphoplasmacytic infiltrates can be seen during histological examination. When lymphoplasmacytic sclerosing pancreatitis and cholangitis occur, the best diagnostic and discriminating test is to test the serum for IgG4 level; it is significantly elevated in this condition. In our case, the histological picture was not specific to this disease at all, the IgG4 level of the patient was normal, so we discarded this diagnosis.

For middle and distal common bile duct carcinomas, pancreato-duodenectomy was the gold standard procedure, but nowadays bile duct segment resection with lymphnode dissection is also established [21]. Complete resection of the tumour with histologically proven negative resection margins ensures the best long-time postoperative survival [22]. The status of the final resected ductal margin is strongly associated with the prognosis of patients: in case of a positive surgical margin, poor long-term survival can be expected [23].

In our case, the intraoperative frozen section proved malignant cells in the samples, but the final histological report came back without any clear malignancy. During the procedure of frozen sectioning, the nuclei of the epithelium cells can be injured: in such a case, it is difficult to discriminate the epithelium for dysplasia or carcinoma in situ [24]. On the other hand, traditional haematoxylin eosin (H&E) stained sections often fail to provide sufficient information to establish the appropriate diagnosis or to make a proper therapeutic decision. The enhancer of zeste homologue 2 (EZH2) is a recently used marker for hepatocellular carcinomas, but it can also be used in the treatment of cholangiocellular carcinomas as it is able to differentiate between neoplastic or benign (or reactive) biliary proliferation [25].

## Conclusion

This case was rare and challenging in every aspect: the malignancy mimicking biliary stenosis in a situs inversus patient required both diagnostic and therapeutic considerations, as well as performing a major surgery in an unusual anatomic situation. At 6 months of follow up, the patient was doing well: the control CT scan did not prove either recurrence or any other complications.

## Supplementary Information

**Additional file 1:** Timeline of diagnostic and therapeutic steps.

## Data Availability

Medical records of the patient are registered in the official medical data system of Semmelweis University.

## Abbreviations

CBD: Common bile duct
CHA: Common hepatic artery
CT: Computer tomography
ERCP: Endoscopic retrograde cholangiopancreatography
EUS: Endoscopic ultrasound
EZH2: Enhancer of zeste homologue 2
GB: Gall bladder
H&E: Haematoxylin and eosin
LGA: Left gastric artery
LHA: Left hepatic artery
MRCP: Magnetic resonance cholangiopancreatography
PTD: Percutaneous transhepatic drainage
SIT: Situs inversus totalis
SMA: Superior mesenteric artery
