# Supplementary material for: Double rarity: malignant masquerade biliary stricture in a situs inversus totalis patient
Source: BMC Surg. 2021 Mar 21;21:153. doi: 10.1186/s12893-021-01155-w (PMC7981884; doi:10.1186/s12893-021-01155-w)
Supplement: Supplementary file 1 — Additional file 1: Timeline of diagnostic and therapeutic steps. [file 12893_2021_1155_MOESM1_ESM.pdf]

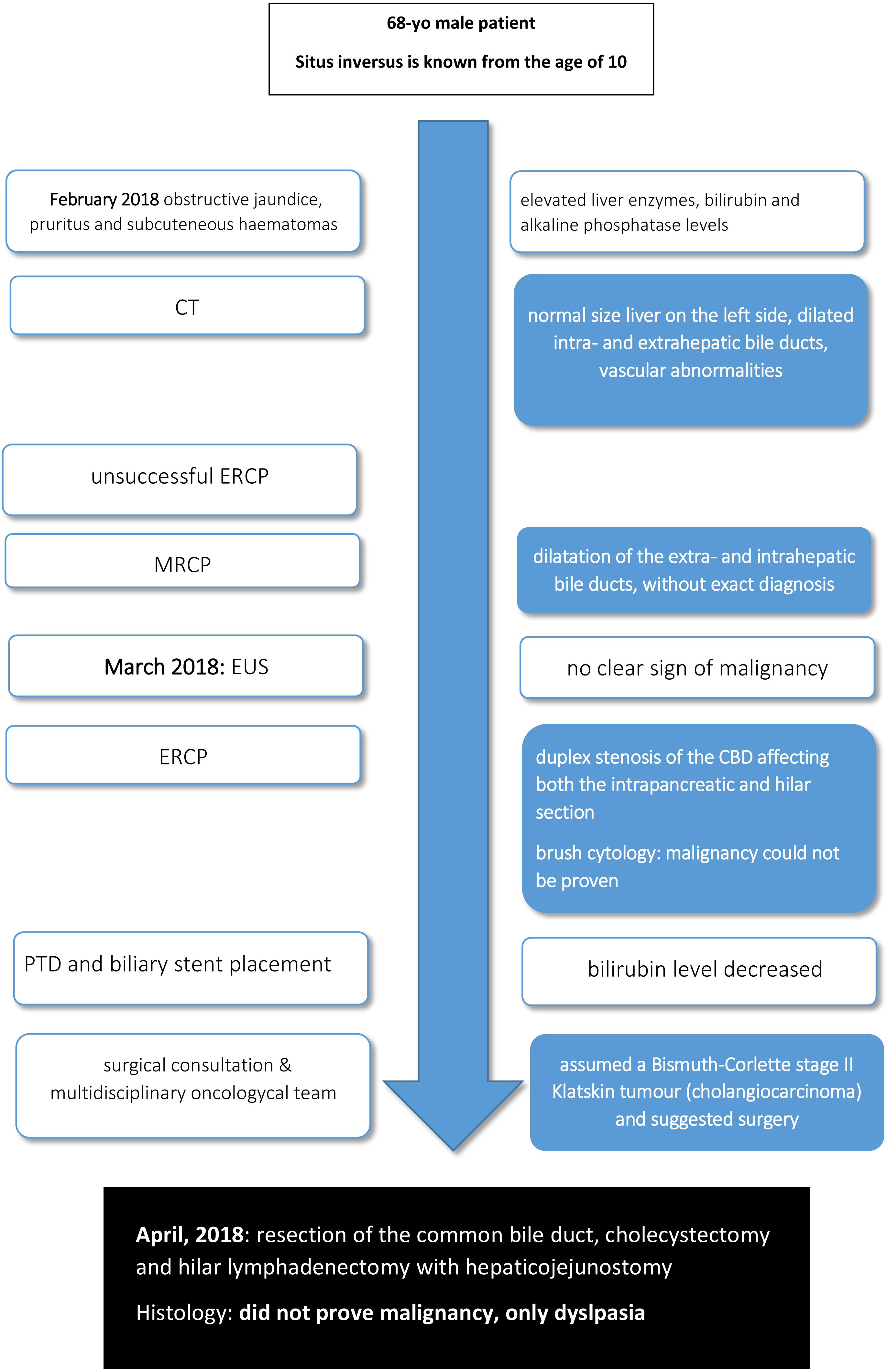
68-yo male patient
Situs inversus is known from the age of 10
February 2018 obstructive jaundice, pruritus and subcuteneous haematomas
elevated liver enzymes, bilirubin and alkaline phosphatase levels
CT
normal size liver on the left side, dilated intra- and extrahepatic bile ducts, vascular abnormalities
unsuccessful ERCP
MRCP
dilatation of the extra- and intrahepatic bile ducts, without exact diagnosis
March 2018: EUS
no clear sign of malignancy
ERCP
duplex stenosis of the CBD affecting both the intrapancreatic and hilar section
brush cytology: malignancy could not be proven
PTD and biliary stent placement
bilirubin level decreased
surgical consultation & multidisciplinary oncologycal team
assumed a Bismuth-Corlette stage II Klatskin tumour (cholangiocarcinoma) and suggested surgery
April, 2018: resection of the common bile duct, cholecystectomy and hilar lymphadenectomy with hepaticojejunostomy
Histology: did not prove malignancy, only dyslpasia
